# Self-Healable and Reprocessable Silicon Elastomers Based on Imine–Boroxine Bonds for Flexible Strain Sensor

**DOI:** 10.3390/molecules28166049

**Published:** 2023-08-14

**Authors:** Peng Wang, Zhuochao Wang, Lu Liu, Guobing Ying, Wenxin Cao, Jiaqi Zhu

**Affiliations:** 1 Department of Materials Science and Engineering, College of Mechanics and Materials, Hohai University, Nanjing 211100, China; liulu201709@163.com (L.L.); yinggb2010@126.com (G.Y.); 2 National Key Laboratory of Science and Technology on Advanced Composites in Special Environments, Harbin Institute of Technology, Harbin 150080, China; 20s118208@stu.hit.edu.cn (Z.W.); zhujq@hit.edu.cn (J.Z.)

**Keywords:** boroxine, imine, self-healing, silicone, sensor

## Abstract

Silicon elastomers with excellent self-healing and reprocessing abilities are highly desirable for the advancement of next-generation energy, electronic, and robotic applications. In this study, a dual cross-linked self-healing polysiloxane elastomer was facilely fabricated by introducing an exchangeable imine bond and boroxine into polydimethylsiloxane (PDMS) networks. The PDMS elastomers exhibited excellent self-healing properties due to the synergistic effect of dynamic reversible imine bonds and boroxine. After healing for 2 h, the mechanical strength of the damaged elastomers completely and rapidly recovered at room temperature. Furthermore, the prepared PDMS elastomers could be repeatedly reprocessed multiple times under milder conditions without significant degradation in mechanical performance. In addition, a stretchable and self-healable electrical sensor was developed by integrating carbon nanotubes (CNTs) with the PDMS elastomer, which can be employed to monitor multifarious human motions in real time. Therefore, this work provides a new inspiration for preparing self-healable and reprocessable silicone elastomers for future flexible electronics.

## 1. Introduction

Polysiloxane elastomers are widely used in strain sensors, seals, biomedical devices, and many other fields because of their unusual properties, including high elasticity, chemical stability, nontoxicity, and biocompatibility. Therefore, they have attracted significant attention recently [1,2]. However, they are vulnerable to damage in their service life because of their relatively weak mechanical properties. And, due to the presence of irreversible cross-linking structures within the system, most current silicone-based materials are unable to reconstruct their networks once damaged. As a result, these failed materials have to be either incinerated or buried, resulting in environmental pollution and resource wastage. Hence, the development of organic silicone materials with self-healing capabilities and recyclability is of great significance in extending their lifetime and reducing energy waste [3].

To date, many self-healing polysiloxane elastomers have been fabricated by incorporating dynamic interactions (such as hydrogen bonds [4,5], π-π stacking [6], ionic interactions [7,8], and metal coordination [9,10]) or reversible covalent bonds (such as Diels–Alder reaction [11,12], disulfide exchange [13,14], and imine bonds [15,16]) into silicon networks. Among them, imine bonds can be generated through the Schiff base reaction between amino and aldehyde groups under mild conditions in the absence of catalysts. What is more, imine bonds exhibit dynamic reversibility, making them suitable for the development of PDMS materials with a self-healing ability and reprocessability [17,18]. For instance, Yu et al. and Wang et al. have demonstrated the potential for imine-modified polydimethylsiloxane (PDMS) to operate as self-healing elastomers [19,20]. In our previous work, a transparent healable PDMS elastomer was prepared successfully by introducing reversible imine bonds into the PDMS networks [21,22]. Due to the presence of dynamic covalent bonds, these elastomers possess excellent self-healing ability and reprocessability. However, the mechanical properties of these elastomers are relatively low. Therefore, it is still a challenge to develop polysiloxane elastomers with good mechanical properties and excellent self-healing properties without external stimulation. It is effective to enhance the mechanical properties of polymers by adding fillers [23,24], fibers, or additives [25,26], but these methods are often tedious and complex. Designing the molecular structure of polymers to enhance their mechanical properties has unique advantages. Recently, a dual cross-linked strategy was proven to be effective in improving strength as well as stretchability [27,28]. Boroxine is produced by a dehydration reaction between boronic acid molecules. Due to its unique triangular molecular structure, it can provide a higher cross-linking density for polymers. Moreover, the formation of boroxine is dynamic and reversible, making it suitable for fabricating mechanically enhanced self-healing materials. Many efforts have been made to design self-healing materials based on boroxine, and these materials show remarkable mechanical properties [29,30]. So, there is a reasonable prospect that combining a high-strength boroxine structure with dynamic imine bonds and constructing a dual cross-linked network have great potential to design polymer materials with both high mechanical strength and outstanding self-healing capacity at room temperature.

Herein, a simple and controllable approach was reported to prepare a dual cross-linked polysiloxane elastomer with self-healing and recyclability via a Schiff base reaction and boronic acid dehydration. The introduction of boroxine into the polymer networks enhanced the mechanical strength of the elastomers. Due to the synergistic effect of the imine bonds and the boroxine, the elastomers could be self-healed at room temperature and showed excellent reprocessability. Moreover, a stretchable and self-healable electrical sensor was fabricated by integrating carbon nanotubes (CNTs) with the PDMS elastomer. The key raw materials used in this study comprised a commercially available amino-modified silicone oil softener. Moreover, the manufacturing process was easily accomplished. Therefore, this research presents an economical and efficient approach to designing self-healing silicone elastomers for flexible strain sensors, thereby offering a novel perspective for the efficient utilization of organosilicon materials.

## 2. Results and Discussion

### 2.1. Synthesis and Characterization of the Dual Cross-Linked PDMS Elastomer

**Figure 1:**
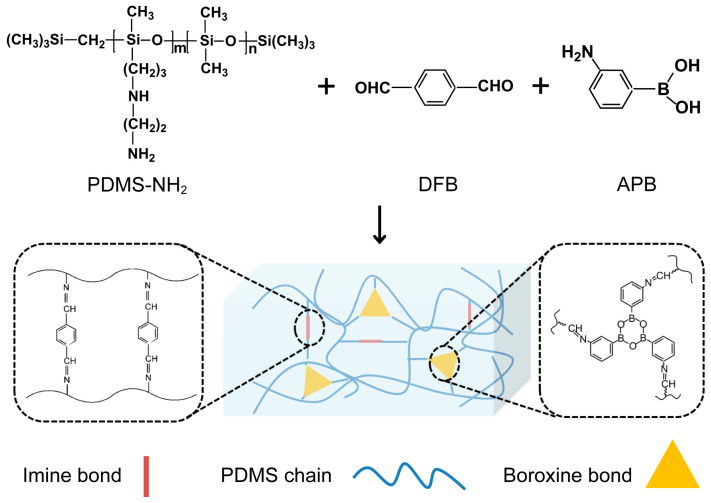
Schematic illustration for the preparation of PDMS elastomer.

The synthesis process and the chemical structure of the elastomer are presented in Figure 1. The main raw material for the synthesis is amino-modified PDMS, with 1,4-diformylbenzene (DFB) and 3-aminobenzenboronic acid (APB) as crosslinking agents. Imine bonds were constructed through a Schiff base reaction between the amino groups on the side chains of PDMS and the aldehyde groups of DFB, which contribute to the self-healing property. Meanwhile, boroxine was formed through a dehydration reaction between the boron hydroxyl groups of APB, which impart toughness to the elastomer. Finally, two dynamic bonds—weak imine bonds and strong boroxine—were introduced into the silicone polymer networks, resulting in a dual-crosslinked self-healing PDMS elastomer. The synergistic effect of dynamic imine bonds and boroxine endowed the elastomer with good mechanical properties and fast autonomous self-healing ability under ambient conditions.

The successful synthesis of the dual cross-linked polymer was confirmed using Fourier-transform infrared spectroscopy (FT-IR) and ^1^H NMR. As shown in Figure S1, the FT-IR spectrum of dual cross-linked elastomer show that characteristic absorption bands at 1623 cm^−1^ appeared, which can be ascribed to the C=N stretching vibration [30]. Moreover, as the concentration of APB increased, the characteristic peak of the imine bond became more prominent. This indicates that more APB was introduced into the polymer network through Schiff base reactions. In addition, two new bands appeared at 746 and 687 cm^−1^, which can be attributed to the absorption peak of boroxine [29]. This suggests the formation of boroxine structures through dehydration reactions between boronic acids from APB. Further evidence from ^1^H NMR spectra is provided in Figure S2. The peaks observed at b (δ = 0.04–0.08 ppm); c (δ = 0.52 ppm); d (δ = 1.27 ppm); e, f, and g (δ = 2.62–2.93 ppm) correspond to the chemical shift in the methyl group (-Si-CH_3_) and methylene group (-Si-CH_2_CH_2_CH_2_-) attached to silicon. In addition, the peaks at h (δ = 7.4–7.8 ppm) and a (δ = 8.37 ppm) are associated with the protons from the benzene ring and imine bond (HC=N), respectively. This indicates that DFB and APB were successfully introduced into the PDMS networks by Schiff base reactions and dehydration reactions with amino PDMS, forming dynamic imine bonds and boroxine structures. All these results suggest that the PDMS elastomers were prepared successfully by introducing imine bonds and boroxine.

Thermogravimetric analysis (TGA) was carried out to determine the composition of the elastomers. As shown in Figure S3, the sample exhibited only one broad weight loss in the temperature range of 300 °C to 600 °C, which corresponds to the breaking and decomposition of the siloxane chains at high temperatures [31], indicating that the resulting polymers are single-component compounds without other impurities. Taking a weight loss of 5% as the thermal decomposition temperature, through calculations, the thermal decomposition temperature of dual cross-linked PDMS elastomer is determined to be 326 °C, which is higher than the PDMS elastomer based on only imine bonds in our previous work [21]. This can be attributed to the higher crosslinking density and stronger bonding energy in the dual cross-linked PDMS elastomer sample. Such a high thermal decomposition temperature indicates that the dual cross-linked elastomers have good heat resistance and thermal stability. Meanwhile, in order to characterize the glass transition temperature (*T_g_*) and thermally amendable characteristic of the PDMS elastomer, DSC analysis was performed via a heating and cooling cycle in a temperature range of −150 °C to 100 °C. As shown in Figure S4, the PDMS elastomer does not exhibit any endothermic or exothermic peaks in the heating and cooling curves, indicating the absence of a melting temperature (*T_m_*) and a crystallization temperature (*T_c_*). This observation suggests that the polymer is in an amorphous state. Additionally, it is also possible that the polymer has a slow crystallization rate, thereby not showing distinct crystallization and melting peaks during the cooling and heating processes. By calculation, the glass transition temperature (*T_g_*) of the dual cross-linked elastomer is about −128 °C, which is close to the PDMS polymer. Such a low *T_g_* promotes the better mobility of polymer chains, leading to chain diffusion, bond exchange, and re-entanglement on broken surfaces [32]. This is beneficial for self-healing behavior.

Rheology tests were performed to evaluate the viscoelastic properties of the self-healing elastomers. It can be seen from Figure S5 that within the frequency range of 0–100 rad/s, as the frequency increases, both the storage modulus (G′) and loss modulus (G″) show a slight increase. Furthermore, the value of the storage modulus of all samples is always larger than the loss modulus, suggesting that the elastic property of the elastomer is predominant over the whole frequency range and that the material primarily undergoes elastic deformation during stretching. Moreover, the viscoelastic properties of elastomers can be affected by the contents of APB. The higher G′ and G″ values were observed with a higher APB concentration, indicating that APB, as a rigid segment introduced into the polymer network through the formation of boroxine structures, can reinforce the interaction of boroxine and increase the cross-linking density. Consequently, this improves the mechanical properties of the elastomers.

### 2.2. Mechanical Properties of PDMS Elastomer

**Figure 2:**
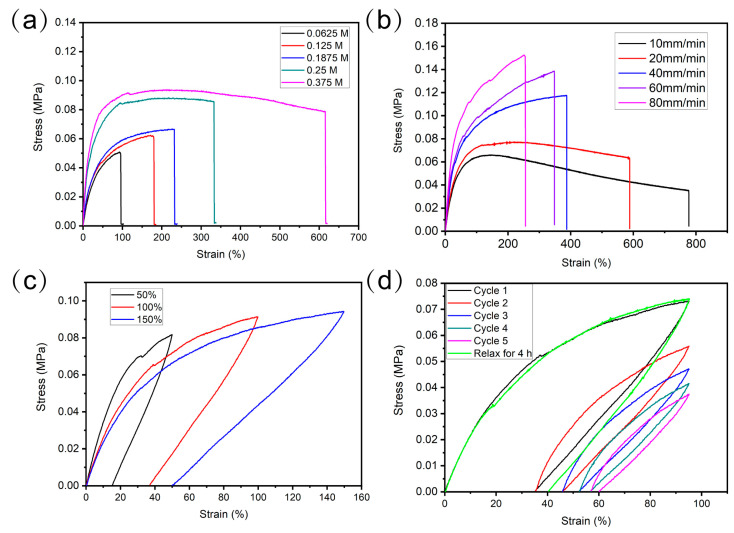
Stress-strain curves of PDMS elastomer (**a**) with different APB concentrations and (**b**) at different stretching speeds. (**c**) Cyclic stress–strain curves of the elastomer under different maximum strains from 50% to 150%. (**d**) Loading–unloading curves of elastomer with different cycles.

In order to evaluate the mechanical properties of the elastomer with different APB contents, tensile tests were performed to obtain the stress–strain curves. As shown in Figure 2a, with an APB concentration increase from 0.0625 M to 0.375 M, the tensile stress and elongation increased from 0.050 MPa and 93.76% to 0.078 MPa and 615.78%, respectively. Coinciding with rheological results, the sample with high APB concentration shows high tensile stress without compromising extension performance, which is very impressive among the reported self-healing PDMS materials. The improved mechanical properties of PDMS elastomers are due to the incorporation of boroxine with imine bonds. In addition, the stretchability was dependent on the stretching speed. As shown in Figure 2b, by increasing the stretching speed from 10 to 80 mm/min, the elongation at break decreased from 776.14% to 254.19%, while the tensile strength increased from 0.0347 MPa to 0.1523 MPa. And, decreasing the sample dimensions could lead to higher stretchability. Such phenomena are typical of elastic polymers. It can be ascribed to the fact that when higher speed is applied, the polymer segmental motion cannot keep up with the external force, and less time is allowed for the displacement, reorientation, and reconfiguration of polymer chain segments. Meanwhile, this shortens the time for dynamic covalent bond breaking and polymer network reconstruction. As a result, the elastomer exhibits thermosetting characteristics, reducing the resistance of polymer chains and leading to lower fracture elongation. On the contrary, at lower stretching rates, polymer chains have enough time to relax and rearrange. The polymer exhibits viscoelastic characteristics. Therefore, the sample shows higher extensibility at lower stretching rates. Moreover, the mechanical strength of polymers is influenced by the gage length during the test (Figure S6). As the gage length increased from 5 mm to 20 mm, the tensile strength and fracture elongation of the PDMS elastomer decreased from 0.12 MPa and 1880% to 0.07 MPa and 371%, respectively. This indicates that reducing the gage length of the material results in higher mechanical performance. The main reason for this observation may be that there are more defects in samples with larger dimensions, making it easier for the polymer to break through defect propagation upon tension. Therefore, with longer gage lengths, the probability of fracture increases, leading to lower fracture elongation and tensile strength of the sample [33,34].

To further understand the characteristics of the hysteresis, cyclic tensile experiments were carried out. As seen in Figure 2c, pronounced hysteresis loops can be observed during the stretching cycle. As the strain increases from 50% to 150%, the residual strain increases from 15% to 50%. This is because the polymer network contains massive sacrificial dynamic covalent bonds, such as imine bonds and boroxine. When a small strain is applied, weaker imine bonds start to break, resulting in energy dissipation. As the strain increases, stronger boroxine structures also begin to break, leading to a significant increase in energy dissipation. Therefore, at higher strains, there is greater energy dissipation, ultimately leading to the breakdown of the cross-linked network. As a result, the elastomer is unable to immediately fully restore its initial mechanical properties after large tensile strains.

Subsequently, multiple cyclic stress–strain tests were performed. As shown in Figure 2d, the tensile stress of the PDMS elastomer gradually decreased and the hysteresis loops became increasingly pronounced with the increase in cycle numbers. This is because some of the dynamic covalent bonds break under tensile stress and cannot fully recover within the range of cyclic stretching. Only a small fraction of sacrificial bonds can participate in the next cycle. Therefore, as the cycle numbers increased, the tensile strength decreased. However, due to the dynamic reversibility of imine bonds and boroxine structures, the ruptured dynamic covalent bonds can be reformed after a certain relaxation period. Hence, the loading–unloading curve nearly overlaps with the first cycle after relaxing for 4 h at room temperature, indicating that the broken dynamic covalent bonds can be completely rebuilt after the 4 h relaxation, and the polymer returns to its original mechanical performance.

### 2.3. Self-Healing Properties and Recyclability of PDMS Elastomer

**Figure 3:**
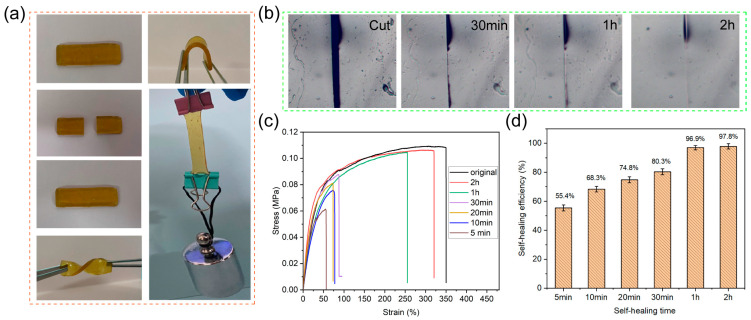
The self-healing properties of the elastomers: (**a**) photographs of the healed samples can withstand twisting, bent, and loading; (**b**) optical microscope images; (**c**) stress–strain curves; and (**d**) self-healing efficiency of the PDMS elastomer healed at room temperature for different times.

The self-healing performance of dual cross-linked elastomer was evaluated. As can be observed in the visual demonstration in Figure 3a, the rectangular elastomer was cut into two separate species, and then, they were brought back into contact for healing for 10 min at room temperature. The specimen after healing can withstand various deformations, like twisting and bending. Notably, the healed sample can hold a weight of 200 g without breaking at the cutting location. From the optical microscopy image in Figure 3b, it can be observed that a wide crack was introduced on the surface of the elastomer using a blade. As the self-healing time increased, the crack gradually became narrower and shallower. The crack almost completely disappeared after healing for 2 h, indicating the excellent self-healing capability of the elastomers. The outstanding self-healing performance of the elastomer can mainly be attributed to the presence of dynamic reversible imine bonds and boroxine structures in the polymer networks. When the two cut pieces were contacted together, the dynamic covalent bonds rebuilt and the polymer chains could undergo diffusion and rearrangement. Ultimately, the elastomer recovered its mechanical properties. Additionally, the self-healing properties were estimated quantitatively by tensile test. The stress–strain curves of the healed elastomer with different healing times are shown in Figure 3c. The original samples exhibited tensile strength and elongation of 0.1082 MPa and 349.50%, respectively, at break. After self-healing for 2 h, the tensile strength and elongation could almost reach to the original samples. It also can be seen from Figure 3d that the self-healing efficiency, defined as the ratios of the tensile strengths of healed and original samples at break, increased from 55.4% to 97.8% with the healing time increased from 5 min to 2 h. All these results suggest that the dual cross-linked elastomers exhibit excellent self-healing performance, which can be achieved at room temperature without the need for light, heat, or radiation intervention.

**Figure 4:**
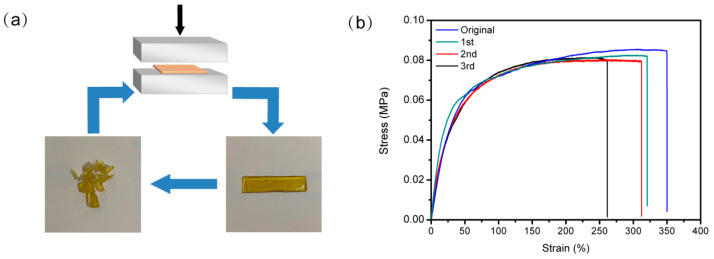
(**a**) Photographs of the samples cut into small pieces and reprocessed by pressing at room temperature. (**b**) The stress–strain curves of the original and regenerated PDMS elastomer.

To examine the reprocessability of the PDMS elastomer, the rectangular samples were first cut into small pieces and then collected together and placed in a polytetrafluoroethylene mold. As shown in Figure 4a, after several hours of compression molding at room temperature, a flexible recycled sample could be obtained again according to the mold shape. The stress–strain curves of the original and recovered samples are shown in Figure 4b. It can be seen that even after reprocessing for three cycles, the mechanical properties of the reprocessed specimens had nearly no decrease, and the tensile strength could be recovered by more than 95%. The results verify the excellent reprocessability of the elastomers, and this method offers a facile way to reprocess polymers under a milder condition rather than heating directly, which conforms to the development trend of environmental protection and resource conservation.

### 2.4. Electrical Performance and Strain Sensor Application

Self-healing materials provide advantages in electronic devices, conductors, and sensors. Herein, a PDMS/CNT composite sensor was fabricated by combining self-healing PDMS with CNT. To evidence the electrical healing characteristic of PDMS/CNT sensors, the rectangular PDMS/CNT sample was connected to a circuit with a light-emitting diode (LED) light and a battery. As shown in Figure S7, initially, PDMS/CNT exhibited good conductivity, and the LED was turned on when a current was applied. Once the sample was cut off with a blade, the circuit was broken, and the LED was turned off. But, when the severed elastomers were brought into contact and allowed to heal for several minutes, the conductive pathway would be restored, and the LED was turned on again, demonstrating the electrical self-healing property of the PDMS/CNT composite.

**Figure 5:**
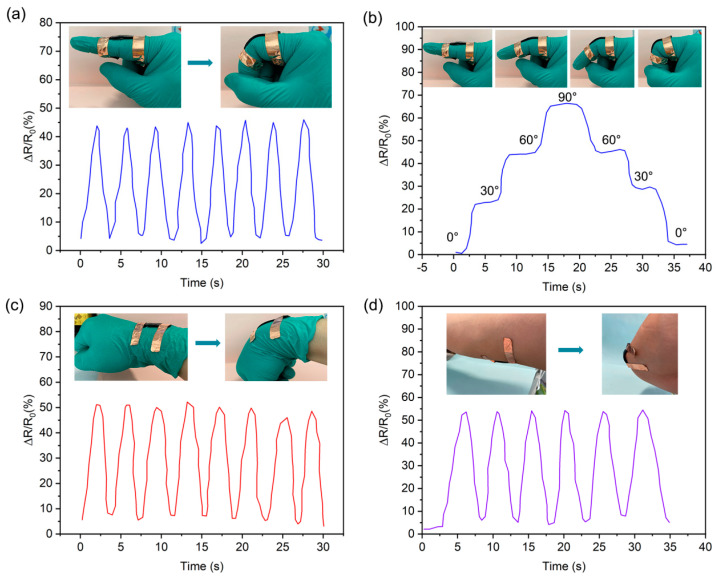
Dynamic response of PDMS/CNT sensor for monitoring various human activities: (**a**) finger bending, (**b**) finger bending with different angles, (**c**) wrist bending, and (**d**) elbow bending.

To evidence that the PDMS/CNT composite is capable of working as a strain sensor for monitoring human joint motions, the self-healing PDMS/CNT sensors were mounted on the finger to detect the activity. The relative resistance changes (Δ*R*/*R*_0_, where Δ*R* is the absolute value of the resistance change with bending and *R*_0_ is the initial resistance without bending) were recorded. As shown in Figure 5a,b, during the process of bending the finger, a clear periodic variation in the relative resistance change signal was observed. Furthermore, as the finger bending angle changed from 0°–30°–60°–90°–60°–30°–0°, the relative resistance change varied to different degrees due to the different deformations of the elastomer. Higher bending angles corresponded to greater deformations of the elastomer, resulting in higher values of relative resistance change. When the finger bending angle was 90°, the Δ*R*/*R*_0_ could reach up to 65%. Furthermore, due to the excellent electrical conductivity stability of the material, the captured sensing signal remained constant when the finger maintained a certain angle, suggesting the stable and effective sensing performance of the assembled sensor. Furthermore, the monitoring capability of PDMS/CNT for wrist and elbow activity was further explored. As shown in Figure 5c,d, when the wrist and elbow repeated bending–straightening motions, the sensor provided repeatable and distinctive electrical signals. During flexion of the wrist and elbow, the conductive self-healing material underwent deformation, disrupting the electronic conduction pathway and causing a sudden increase in resistance, resulting in an increased relative resistance change. As the resistance of the wrist and elbow returned to the initial state, a conductive pathway was re-established and reached its original status. Thus, the relative resistance became zero. The phenomenon is consistent with the results in Figure 5a. Based on this, it is believed that the prepared PDMS/CNT elastomer can be used as a wearable strain sensor to monitor various human motions in real time.

## 3. Materials and Methods

### 3.1. Materials

Amino-modified PDMS (XIAMETER OFX-8040 fluid, viscosity: 800–5000 cPs, Nitrogen content: 0.32–0.42%) was purchased by Dow Corning Corporation. 1,4-diformylbenzene (DFB) was obtained from Shanghai Macklin Biochemical Co., Ltd. (Shanghai, China). 3-Aminophenylboronic acid (APB) was bought from Aladdin Chemical Ltd. (Shanghai, China). Toluene was supplied by Sigma-Aldrich (St. Louis, MO, USA). Multi-walled carbon nanotube (CNT; diameter, 30–50 nm; length, 10–20 μm) was purchased from Nanjing XFNANO Materials Tech Co., Ltd. (Nanjing, China). 1-pyrenecarboxaldehyde (PA) was purchased from Aladdin. All chemicals were used as received without further purification.

### 3.2. Preparation of Dual Cross-Linked Self-Healing PDMS Elastomer

Firstly, different amounts of 3-Aminophenylboronic acid (APB) were dissolved in toluene and stirred for 6 h. Then, amino-modified PDMS (PDMS-NH_2_) was added to the APB toluene solution. After being stirred for 2 h at room temperature, a certain amount of DFB toluene solution (0.075 mol/L, the concentration was determined according to our previous work) was added to the above solution. After several minutes, a cross-linked organogel was obtained. The polymer mixture was placed in a fume hood for 12 h and further vacuum dried at 60 °C for 6 h to remove the residual solvent. Finally, the dual cross-linked PDMS elastomers were prepared by mold pressing in a polytetrafluoro-ethylene (PTFE) template.

### 3.3. Fabrication of PDMS/CNTs Sensor

Firstly, CNTs were functionalized with PA according to the reported literature with slight modification [35]. Briefly, 100 mg of CNTs was dispersed in 20 mL of toluene solution containing 20 mg of PA using ultrasonication for 2 h. After stirring for 12 h, the mixed solution was centrifuged and washed with ethanol. The functionalized CNTs were obtained after drying in a vacuum at 60 °C for 4 h. PDMS/CNT nanocomposites were prepared by mixing the functionalized CNTs during the cross-linking process. Typically, a certain amount of APB was dissolved in a toluene solution. Then, PDMS-NH2 and a specified amount of functional CNTs were added into the solution and stirred at room temperature for 4 h. Subsequently, the DFB toluene solution was added into the above solution to form cross-linked polymer networks. After the solvent evaporated, the PDMS/CNT composite elastomers could be obtained.

### 3.4. Characterization

Fourier-transform infrared (FT-IR) spectra were recorded on a PerkinElmer Frontier. Each measurement included an average of about 32 scans from 4000 to 400 cm^−1^. ^1^H-NMR spectra were obtained using a Bruker 300 MHz spectrometer with CDCl_3_ as the solvent. Thermal gravimetric analysis (TGA) was carried out using a synchronous thermal analyzer (STA449F3, Selb, Germany) by heating the samples from room temperature to 800 °C at a heating rate of 10 °C/min under the protection of a nitrogen atmosphere. Differential scanning calorimetry (DSC) measurements were conducted on a NETZSCH STA449F3 instrument at a heating rate of 10 °C/min from −140 to 100 °C under a nitrogen atmosphere. Rheological properties were measured on an AR2000ex rheometer (TA Instruments, New Castle, DE, USA) by using 25 mm plates with a parallel-plate geometry. Mechanical properties were measured on rectangular specimens (40 mm × 10 mm × 2 mm) using a universal electronic tension testing machine (Instron 5944, Norwood, MA, USA) with a strain rate of 20 mm/min at room temperature. The self-healing properties of the crack on the polymer were observed using optical microscopy (WMP-6880, Shanghai Wumo Optical, Shanghai, China). The electrical properties of sensors were measured by a power supply (RAC-5V-100A).

### 3.5. Evaluation of Self-Healing Properties

The original elastomer was cut in half using a blade, and the severed sections were reconnected at room temperature for different times (5 min, 10 min, 20 min, 30 min, 1 h, and 2 h). The mechanical properties of the repaired samples were tested using a universal tensile testing machine. The tensile strength and elongation of elastomers healed for different times were obtained from the tensile test. The self-healing efficiency (*HE*) of the elastomer was calculated as follows (Equation (1)):

(1) $$HE=\frac{\sigma_{h}}{\sigma_{0}}\times 100\%$$

where $\sigma_{0}$ and $\sigma_{h}$ correspond to the tensile strength for the original and healed samples, respectively.

## 4. Conclusions

In summary, a self-healable and reprocessable PDMS elastomer was successfully developed through a Schiff base reaction and boronic acid dehydration. The incorporation of boroxine into the polymer networks enhanced the mechanical strength of the elastomers. The integration of dynamic covalent imine bonds and boroxine resulted in excellent self-healing properties, with a maximum healing efficiency of up to 97.8%. Meanwhile, the dynamic dual cross-linked network made the material recycle several times without sacrificing its mechanical property. Finally, a self-healable electrical sensor was fabricated by embedding CNT in the self-healing PDMS matrix, which can be used to monitor various human motions in real time. This work provides inspiration for fabricating self-healing silicone elastomers and offers a promising candidate applied in soft robots, motion monitoring, and other fields.

## Data Availability

Not applicable.

## Supplementary Materials

The following supporting information can be downloaded at: https://www.mdpi.com/article/10.3390/molecules28166049/s1, Figure S1: FT-IR spectra of PDMS elastomer with different APB contents; Figure S2: ^1^H NMR spectra of PDMS elastomer; Figure S3: TGA curves of PDMS elastomer; Figure S4: DSC traces of PDMS elastomer; Figure S5: (a) Storage modulus (G′) and (b) loss modulus (G″) against frequency for PDMS elastomer with different APB concentrations; Figure S6: Stress–strain curves of the elastomer with different gage lengths; Figure S7: The electrical healing process of PDMS/CNT elastomer.

## Sample Availability

Samples of the compounds are available from the authors.
