# Supplementary material for: Self-Healable and Reprocessable Silicon Elastomers Based on Imine–Boroxine Bonds for Flexible Strain Sensor
Source: Molecules. 2023 Aug 14;28(16):6049. doi: 10.3390/molecules28166049 (PMC10458376; doi:10.3390/molecules28166049)
Supplement: Supplementary file 1 [file molecules-28-06049-s001.zip › molecules-2534627-supplementary.pdf]

Supporting information

Self-healable and reprocessable silicon elastomers based on imine-boroxine bonds for flexible strain sensor

Peng Wang [1,*,†], Zhuochao Wang [2,†], Lu Liu [1], Guobing Ying [1], Wenxin Cao [2,*], and Jiaqi Zhu [2]

[1] Department of Materials Science and Engineering, College of Mechanics and Materials, Hohai University, Nanjing 211100, P. R. China

[2] National Key Laboratory of Science and Technology on Advanced Composites in Special Environments, Harbin Institute of Technology, Harbin 150080, P. R. China

* Correspondence: wangpeng8917@126.com (P.W.); caowenxin@hit.edu.cn (W.C.)

† These authors contributed equally to this work.

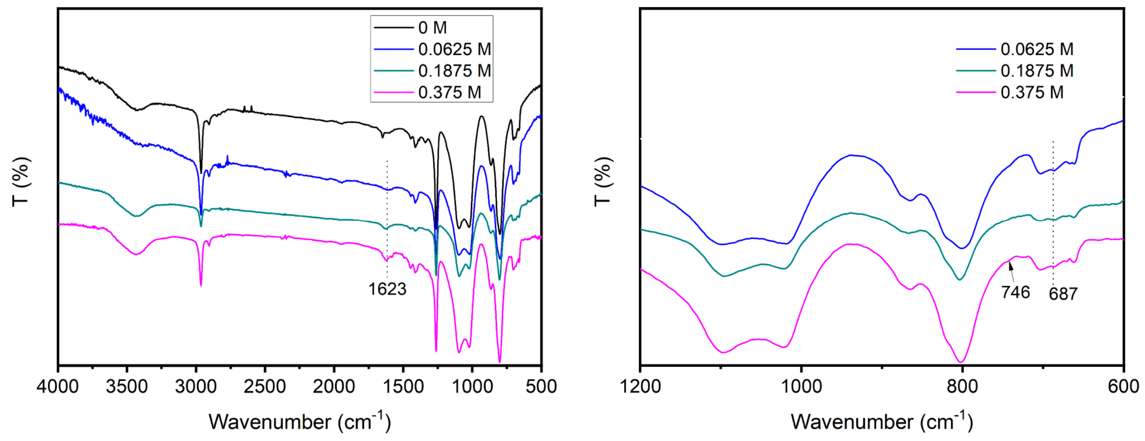

Figure S1. FT-IR spectra of PDMS elastomer with different APB content.

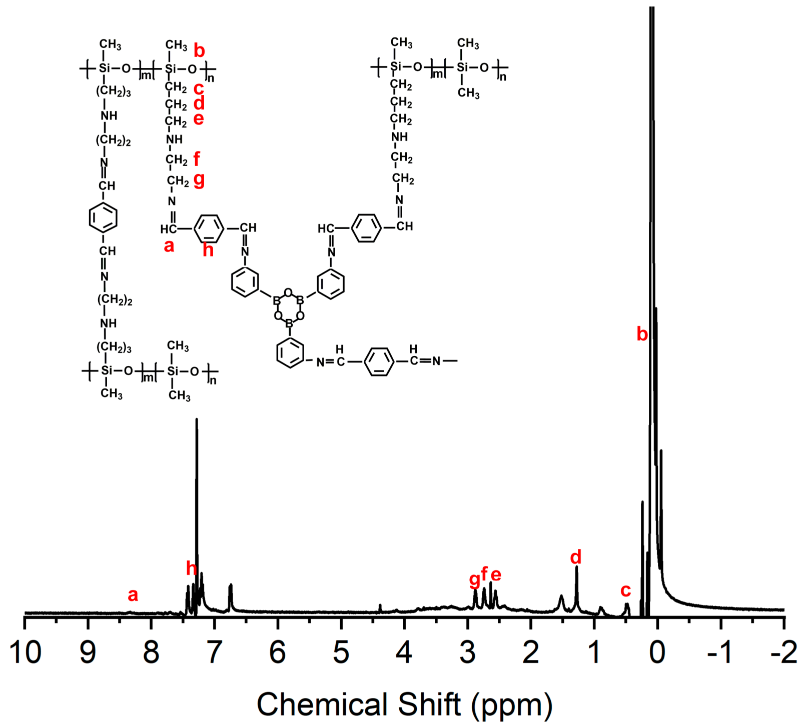

Figure S2. ^{1}H NMR spectra of PDMS elastomer.

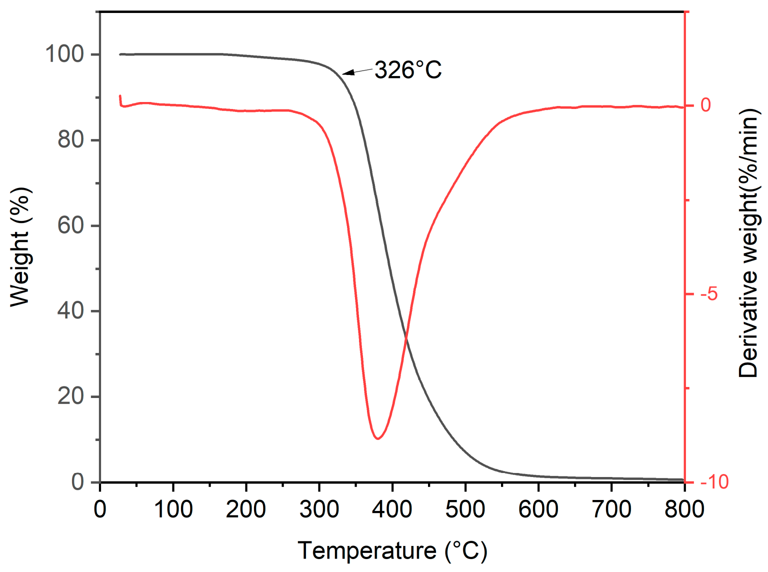

Figure S3. TGA curves of PDMS elastomer.

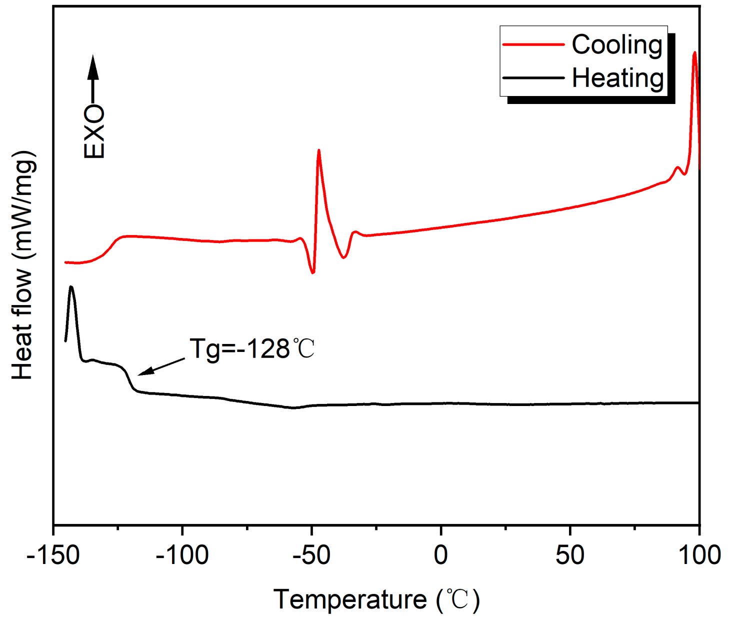

Figure S4. DSC traces of PDMS elastomer.

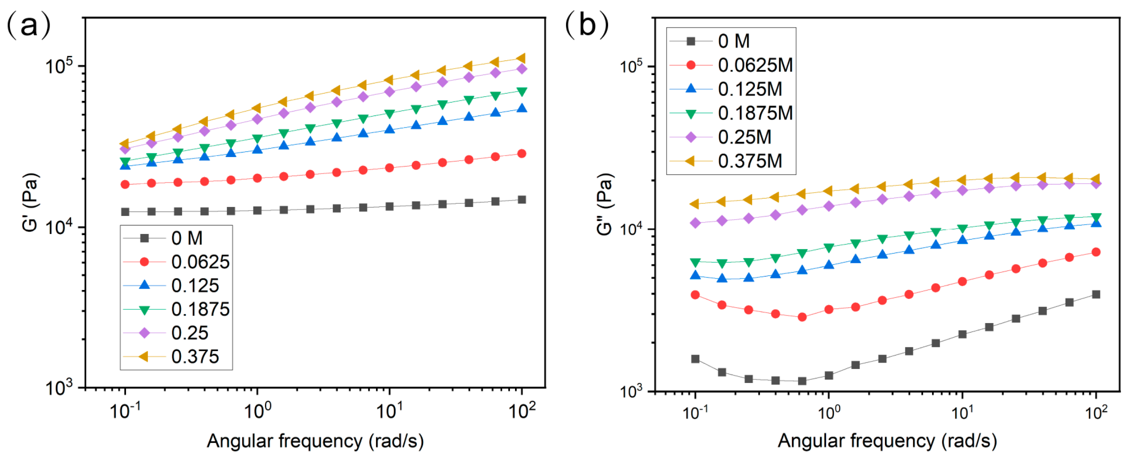

Figure S5. (a) Storage modulus (G′) and (b) loss modulus (G″) against frequency for PDMS elastomer with different APB concentration.

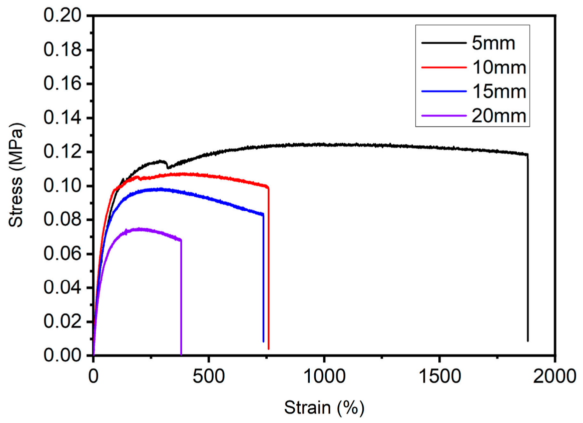

Figure S6. Stress-strain curves of the elastomer with different gage length.

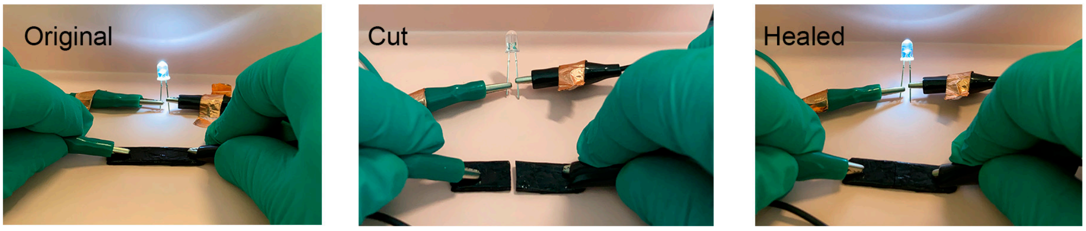

Figure S7. The electrical healing process of PDMS/CNT elastomer.
